# Think Globally Act Locally: The Case for Symphysiotomy

**DOI:** 10.1371/journal.pmed.0040071

**Published:** 2007-03-27

**Authors:** Douwe Arie Anne Verkuyl

## Abstract

When expatriate doctors from developed countries working in sub-Saharan Africa suggest to the local doctors and midwives that symphysiotomies should sometimes be done, they are silenced neither with quotations from the medical literature nor with tales of patients seen, but with: “If symphysiotomies are such good operations why don't you perform them at home?” Here is why.

## Abstract 2

Although symphysiotomy is rarely taught to trainee obstetricians, the operation can benefit women in obstructed labour, and their offspring, argues Verkuyl.

Symphysiotomy is an operation that is done to increase the size of the pelvic outlet to permit vaginal delivery of a baby. The procedure involves surgically dividing, under local anaesthesia, the cartilage of the symphysis pubis. The skin incision is 1.5–3 cm long. Symphysiotomies, like instrumental deliveries, are typically performed in the labour ward and not the operating theatre. Most women walk with the help of a walking frame/chair two to four days after the operation, and 95% can be discharged from hospital within two weeks (Box 1, Figure 1).

**Figure 1:**
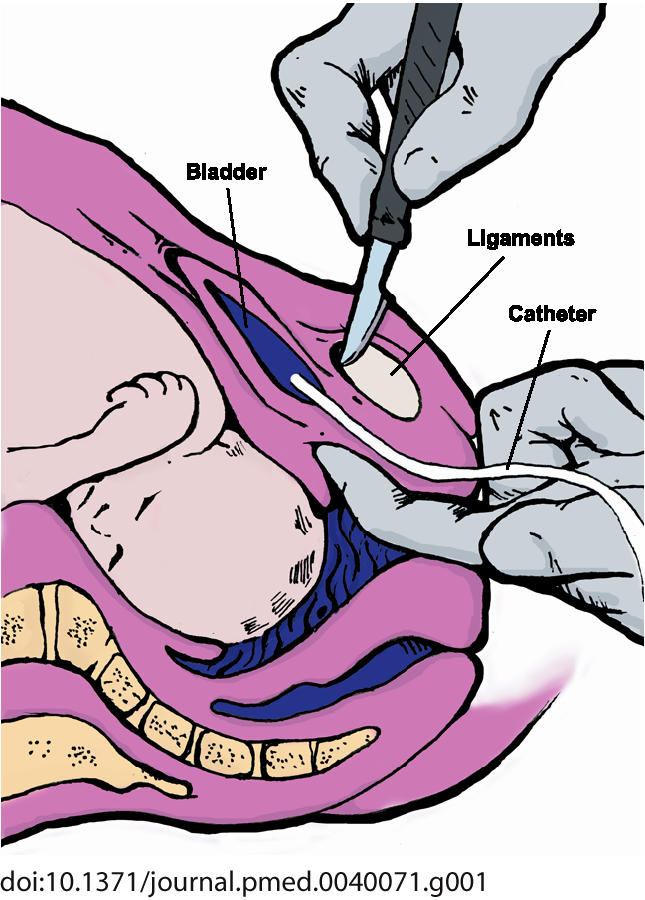
Dividing the Cartilage During Symphysiotomy (Illustration: Anthony Flores, derived from an image at http://www.who.int/reproductive-health/impac/Images_P/fig96dividingm4copy.gif).

**Box 1. Symphysiotomy**

- Performed in (nearly) second stage of labour to temporarily enlarge the pelvis 1–3 cm when there is a mechanical problem.
- Contra-indication: Dead foetus, incomplete dilatation of the cervix, non-longitudinal lie.
- Needed: Local anaesthesia, knife (scalpel with blade 21 or 22 is fine), catheter, vacuum extractor, and good support for the legs to prevent abduction of more than 40° per leg.
- Damage inflicted: Incision 1–3 cm skin and sub cutis over symphysis and severance of the cartilage between the pubic bones.
- Operation takes 2–3 minutes. Women are able to walk, at first painfully, after 2–14 days.
- Scar tissue between the pubic bones will permanently enlarge the pelvis somewhat.
- Side effects: More and longer postoperative pain than with a caesarean section. In the longer term stress incontinence is sometimes experienced (less frequent than after normal vaginal deliveries in Europe) or pain over the symphysis or in the sacroiliac joints. Problems with walking sometimes occur, but are rare [15].
- History: First described in France in 1777. Performed extensively in twentieth century, especially in Catholic countries such as Ireland and Argentina where every contraceptive method, even for medical reasons, other than total abstinence was forbidden by the Catholic Church until 1951. This made multiple pregnancies inevitable and dangerous for women with a small pelvis and a healthy husband [7]. Symphysiotomies were the only alternative to caesarean sections for such women, given that contraception and of course divorce were not options.

## Why Symphysiotomies Have Fallen Out of Favour

In many African hospitals symphysiotomies are no longer performed because doctors believe that, since developed countries can do without them and still achieve excellent obstetric outcomes, this operation is obsolete and Africa will soon “catch up”. Presently many obstetricians in African cities have a private practice and they manage their private patients in conditions that approximate those of a Western setting. Most breech presentations, for example, are delivered by caesarean section (CS). These obstetricians' private patients live in town, have fewer children than rural women, and can be trusted to return for the next (abdominal) delivery, provided political, social, or financial upheavals (such as those related to HIV/AIDS) have not affected their lives too much.

Besides, elective CSs are convenient because they do not interrupt scheduled clinic sessions or prevent doctors from sleeping, and they keep eager lawyers at bay. Naturally, these role models teach the same high standards of care that they apply in their own private practices in the academic hospitals, unlike—suspiciously, in the eyes of local doctors and midwives—the previous generations of consultant obstetricians who would perform symphysiotomies on poor, indigenous patients only.

The academic salaries given to doctors up to about 30 years ago were typically sufficient for a consultant obstetrician to survive on without needing to resort much to private practice. Therefore in a teaching hospital at least one of the senior consultants would be physically present most of the time—crucial for teaching symphysiotomies, which are never elective operations. CSs, on the other hand, can often be taught step by step at convenient hours.

Today, young doctors not trained in (and often brainwashed against) symphysiotomies are posted to the districts without the seniors, intensive care units, experienced theatre staff, blood banks, ultrasound back up, or specialist anaesthetists more or less taken for granted in their training hospitals. These doctors are confronted by very high maternal mortality figures. They often cannot be sure if any doctor will be around in three years time to assist a woman in labour whose uterus they have scarred. They themselves (as well as their solitary nurse anaesthetist) are perhaps in the process of applying for better paid employment by aid organisations or abroad. Who would blame them? Indeed, the World Heath Organization expects an even more serious lack of doctors in Africa in the future.

## Symphysiotomies Are Life-Saving

Björklund recently reviewed the literature on symphysiotomy published in the twentieth century [1]. There were three criteria for including a study in the review: (1) the cases reported had to be consecutive; (2) the studies had to include an acceptable description of methodology; and (3) the study size was set at a minimum of 25 cases for analysis of maternal and foetal mortality.

Comparing symphysiotomies to CSs in circumstances that are now only present in developing countries, Björklund found that: maternal mortality is much lower with symphysiotomies; the short-term complications are less serious; and babies do not do worse as a result of symphysiotomy. And, although there may be a higher rate of long-term minor and moderate side effects with symphysiotomy, the operation results in far fewer subsequent CSs. Björklund concluded: “If valid conclusions can be drawn from one hundred years of retrospective studies, there is considerable evidence to support a reinstatement of symphysiotomy in the obstetric arsenal, for the benefit of women in obstructed labour and their offspring”.

Similar results were reported in 2004 from one hospital in Nigeria where 1,013 consecutive symphysiotomies were performed over an 18-year period [2]. There was only one maternal death (from massive pulmonary embolism), one iatrogenic vesico-vaginal fistula (VVF), and two women had long-term gait problems. In one year, 1985, the hospital had a symphysiotomy rate of 6.6% and over the entire 18-year period a rate of 3.7%.

Onah and Ugona surveyed women in the same region of Nigeria to find out their preferences for symphysiotomy versus CS [3]. Of 777 pregnant women who were familiar with CS and symphysiotomy, and who were asked which they would prefer should the need arise at the end of their pregnancy, 63.1% said symphysiotomy. However, most obstetricians in Nigeria still refuse to perform this operation [3].

A recent study in *The Lancet* found an astoundingly high maternal mortality ratio in one remote area of Afghanistan: one per 15 live births (95% confidence interval: 1/12.5–1/20, a life-time risk of 1:3–4 women) [4]. The most frequent cause of death was obstructed labour, and concerned mainly women in their first pregnancy. Of the babies born alive, 70% died if the mother died. The availability of relatively low-tech symphysiotomies would be an intermediate way to improve this dire situation.

## Breech Deliveries: Symphysiotomy, Caesarean Section, or Accepting Higher Perinatal Mortality?

In most countries in the Western Hemisphere the pendulum is swinging again towards the aphorism “once a caesarean section, always a caesarean section”. On top of that, breech presentations at term (3% of all deliveries) are delivered by CS. It follows that if women have two children, 4.5% of all deliveries would be by CS because of present or previous breech presentations.

Replicating this approach in sub-Saharan Africa, with four children per woman, would mean that more than 7% of all deliveries would need a CS just for breeches past or present. In the United States, with an overall CS rate of 29%, “only” 15% (4.5/29) of all CSs are breech related. Transplanting the US strategy for breech presentations and previous CSs to those areas of Africa where health services are provided by district and mission hospitals would mean that the current overall CS rate would have to quadruple just to accommodate the breech-related CSs.

The Term Breech Study, a randomized trial of 2,088 women with a singleton foetus in a frank or complete breech presentation, found that perinatal mortality and neonatal morbidity was significantly lower with planned CS than with planned vaginal birth [5]. Following the publication of this study, the CS rate in the Netherlands for breech presentations has risen from 50% to 80%. This was an abrupt change, while other factors affecting perinatal mortality remained more or less stable. Data registration on a national level made it therefore possible to conclude that around 12 extra babies with a breech presentation survived annually because of the increase in the CS rate. It could be worked out that 175 extra CSs were performed for the survival of each of those babies [6].

However, problems associated with a CS do not end with the first operation. Further analysis (see Box 2) indicates that in fact a total of 221 CSs, including repeat CSs, (plus 62 trials of scar [an attempt at a vaginal delivery after a previous CS]) have to be performed to save one baby. This calculation includes the operations needed to compensate for the extra foetal loss in future pregnancies. These procedures will kill an estimated 0.012 mothers (84 babies saved for the price of one mother). For many women (and maybe their husbands/partners) this risk may seem reasonable. However, among gynaecologists in the Netherlands blessed with developed world facilities and little litigation pressure, opinions differ widely about the real benefits, if any, of performing so many (repeat) CSs for breech presentations.

**Box 2. Assumptions about Breech Presentation, Caesarean Section, and Symphysiotomy**

- Term vaginal breech deliveries in hospital are equally risky for babies in Africa, Asia, and Europe. Breech deliveries are equally distributed over all parities. Women in Europe have two, those in the relevant areas in Africa and Asia four deliveries.
- If thresholds for CS in breech presentations are lowered so that the CS rate rises from 20% to 50%, one additional baby will initially survive for 29 extra CSs. If the rate goes up from 50% to 80%, one extra baby for 175 CSs survives (the law of diminishing returns). In the entire 20%–80% range this means one baby saved for 50 CSs.
- The next delivery after one previous CS is an elective CS in 30% of women and a trial of scar for 70% of women (two in seven of these trials fail and become a CS). Women who have a successful trial of scar will have another trial of scar with subsequent deliveries. But twice a CS, always a CS.
- In the Netherlands, the *extra* CS-related maternal mortality is one woman per 25,000 CSs. One in 20,000 women and one in 5,000 babies die from a trial of scar [16].
- In Africa and some parts of Asia, the maternal mortality caused directly by a CS is one per 150 CSs [17]. If mothers die, half of their babies die directly or later because of lack of breast-feeding and care. Trials of scar will sometimes occur at home, during transport, or in poorly equipped/staffed health facilities, hence maternal and perinatal mortality is at least one per 100 trials of scar.
- With a *perceived* entrapped after-coming-head in breech deliveries, CS is not an option and on average three symphysiotomies will be needed to prevent the death of one baby.
- In failed instrumental deliveries one symphysiotomy will prevent one foetal death, while a speedily organised CS in a well equipped and staffed theatre will also prevent one foetal death but might cause problems in the future exponentially related to the number of future caesareans [18].
- Symphysiotomies do not cause maternal mortality. In a systematic review of the literature Björklund found no fatal maternal complications in the period after 1950 (the era of antibiotics) directly caused by symphysiotomies [1]. Such lethal complications are probably as rare as are fatalities caused by episiotomies.

In many sub-Saharan African hospitals the direct maternal mortality associated with CS is higher than one per 175 operations. This means that if sub-Saharan Africa were to copy the new Dutch approach, there would be more maternal deaths than babies saved. If 50% of the breech babies delivered by CS die when their mothers die, then one has to perform more than 350 CSs to save one extra baby. Add the mortality of repeat CSs and trials of scar, and it can be calculated (see Box 2) that if women in Africa have four children and the CS rate for breeches should also rise from 50% to 80%, 2.6 babies plus 3.6 women die per successful rescue of one baby.

The higher the total fertility rate (TFR), the more repeat CSs and trials of scar there will be. But even in the case of “only” three deliveries per woman, when the CS rate for breech presentations rises from 20% to 80% for first deliveries, 19 women will eventually die for the survival of one extra baby. It follows that the management of breech presentations currently in fashion in much of the West is not yet the solution for much of the world. Therefore the real dilemma is deciding between: (1) accepting breech-related perinatal deaths (perhaps one a year in the average district hospital with 2,000 deliveries) or (2) performing the occasional symphysiotomy to prevent such deaths.

After symphysiotomy, as opposed to after a CS, a woman's pelvis is somewhat larger. In a subsequent pregnancy, failure to reach a well equipped and staffed hospital in time for the next delivery would result less often in a disaster for patients with a larger pelvis and without a uterine scar.

## Indications for Symphysiotomy and Geographic Variation

There is a large difference in the potential benefits from symphysiotomy in sub-Saharan Africa and parts of Asia compared to Europe. In Europe, TFRs are low, and in many parts of Europe there is a high ratio of obstetricians to patients. In addition, common indications for symphysiotomies in sub-Saharan Africa—such as failed instrumental delivery with no rapid access to theatre and anaesthetist, or neglected, obstructed, and infected labour [7] with a live baby, or severe anaemia and no (safe) blood available—are uncommon in Europe.

Table 1 shows the difference in impact on maternal and perinatal mortality of a symphysiotomy in Africa and the developed world. The key to the table is to understand that it is postulated (see Box 2) that in breech deliveries three symphysiotomies are needed on average to save one baby's life. This low threshold for symphysiotomy is recommended to prevent damaged babies as well as perinatal deaths. It is assumed that, in the 50%–80% CS rate range for breeches, 175 CSs have to be done initially to prevent one baby's death and avert most non-fatal foetal damage. Furthermore, 262.5 repeat CSs and 297.5 trials of scar have to be added for nullipara in Africa. One symphysiotomy prevents therefore 146 CSs ([175 + 262.5 = 437.5]/3) and 99 trials of scar (297.5/3). The figures for Europe are 88 CSs ([175 + 87.5 = 262.5]/3) and 41 trials of scar (122.5/3). All these procedures have mortality costs as postulated in Box 2 and recorded in Table 1.

As mentioned above, the increase in CS rate for breech presentations from 50% to 80% in the Netherlands (TFR 1.71 in 2005) probably saves the lives of around 12 babies on an annual basis [6]. With the number of obstetricians in the Netherlands (761 in 2004), this works out as one baby in 63 years per obstetrician. If three symphysiotomies (instead of 175 CSs) are needed to prevent one perinatal death, an obstetrician would need to perform a symphysiotomy for this indication once in 21 years. Even if there was a return to the CS rate for breeches of 20%, prevalent in the years when CSs were nevertheless not as dangerous as in Africa presently, then one obstetrician would be expected to do only one symphysiotomy in three years (see Box 2).

**Table 1:**
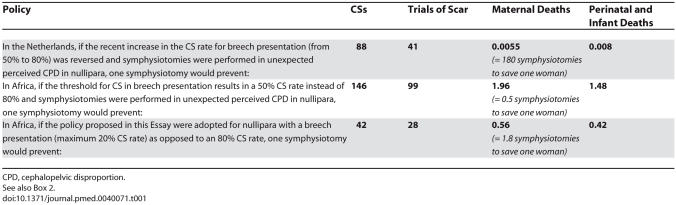
Caesarean Sections, Trials of Scar, and *Extra* Mortality Prevented per Symphysiotomy, if Perceived Cephalopelvic Disproportion in Breech Presentations Were *Treated* by Symphysiotomy instead of *Prevented* by a High CS Rate in the Netherlands and Sub-Saharan Africa (or Afghanistan)

Not only are the indications for symphysiotomy rare in developed countries, but the cases that might benefit from symphysiotomy—mainly obstructed after-coming-head and failed instrumental delivery in a woman unfit for an urgent CS—are such dire emergencies, that it is hardly a suitable opportunity to teach the procedure or even for an obstetrician to maintain a rarely used skill. Indeed these emergencies are nearly always prevented by performing CSs for any case that deviates from normal before it ever reaches the situation where symphysiotomy has become the best option. In contrast, district hospital doctors in Africa could maintain their proficiency with three to ten symphysiotomies annually for all indications, most of which are not seen in developed countries.

## Symphysiotomy for Failed Vacuum Extraction

There are consistent, worrying reports from Africa about long intervals between the decision to perform an *emergency* CS and delivery. Studies from Tanzania, Nigeria, and a tertiary hospital in South Africa showed a mean interval of 3.5, 4.4, and 1.9 hours respectively [8–10]. It is likely that hospitals with even worse results do not publish them. Sometimes, after a failed vacuum extraction, a woman has to be transported to another hospital for a CS. In Africa and Asia delays of up to 24 hours are common in situations where the family has to give permission and/or borrow money, hunt for gloves, a catheter, antibiotics, blood, or even for a female doctor to perform the surgery. Women may develop vesico-vaginal fistulas while waiting for a CS [11].

In some government hospitals, around 5%–10% of the CSs performed for obstructed labour and/or foetal distress result in perinatal death because of theatre delay [8,11,12]. Often nobody is blamed because the decision for performing a CS is seen as the ultimate service a doctor can render and delay is “one of those things” or “God's will”.

If on the other hand an attempt to perform a vacuum extraction fails, and then the waiting time for a CS results in a dead baby (awareness of long theatre delays also encourages of course too much traction), doctors are held responsible. Consequently in many African hospitals the vacuum extractor is rarely if ever used. Midwives are known to hide the apparatus, and often it is not in working condition. If one in ten vacuum extractions fails, and theatre delays and associated desperate pulling kill a number of those babies, and for these reasons instrumental deliveries are not performed, then the solution is obviously to perform vacuum extractions if there is a reasonable chance of success, and perform a symphysiotomy if an extraction happens to fail.

One symphysiotomy after such a policy change prevents initially ten CSs, and later the repeat operations. Nobody can maintain seriously that the complications of one symphysiotomy are worse than those—in nullipara—of 25 CSs plus 17 trials of scar. In fact, three symphysiotomies (preventing 75 CSs plus 51 trials of scar) will in this situation prevent the death of one woman (see Box 2). In those rich countries where vacuum extractions are always attempted if they seem feasible, and where a prompt CS can be done if this turns out to be a miscalculation, 1.5 CSs and 0.7 trials of scar would be prevented, in nullipara, by one symphysiotomy. In that situation, 10,500 symphysiotomies would be needed to save the life of one woman. Furthermore, based on the assumptions in Box 2, one extra baby would survive in Africa for every four symphysiotomies performed. In Europe 7,140 symphysiotomies would be needed to accomplish this feat.

In a typical African district hospital five symphysiotomies for a failed vacuum extraction could be performed annually. These five operations performed on all parities would, it can be calculated, prevent one maternal and nearly one foetal death on average. But these prevented deaths might not be obvious because they could occur years after the initial operation: for example, a woman plus the baby inside her not dying of a ruptured scar, three pregnancies after her first delivery which ended in a symphysiotomy instead of in a CS. On top of that, for these five symphysiotomies in our typical district hospital 45 other women were spared a CS (and later the repeat operations) and they also did not even have a symphysiotomy because the reintroduction of the occasional symphysiotomy made performing vacuum extractions on them again an attractive option. How do you register that a woman, who did not have a CS or a symphysiotomy, does not die in ten years time because of a ruptured uterus after the only transport in her village at that future date had broken down?

## A Policy Proposal

Based on the analysis presented so far, I believe that doctors in sub-Saharan Africa should be taught how to perform symphysiotomies, initially with the help of plastic and metal models as used in the United Kingdom [13], and later in clinical practice. External versions in breech presentations detected before labour should, of course, be tried towards the end of pregnancy (a high HIV prevalence might be a contraindication to version of breeches in untested women and for those known to be HIV positive).

Many caesarean sections are prevented, in the case of cephalic presentations, by the judicious use of oxytocin to augment inadequate contractions. In trials of labour in breech presentations, many obstetricians are reluctant to use this drug, thereby increasing the CS rate. This reluctance is based on the fear that sometimes assumed lack of expulsive force could mask cephalopelvic disproportion. Oxytocin might then help force the baby's breech through a marginal pelvis, setting the stage for an entrapped after-coming-head. Oxytocin could be used as for cephalic presentations if one accepts that one may occasionally have to perform a symphysiotomy if the above scenario ensues.

I suggest that during the second stage of labour one aims, if there is steady progression, for an assisted breech delivery, but prepares for a symphysiotomy, which has a better outcome than a difficult forceps delivery. If progress occurs but is slow and/or the feet of the baby look very large, the symphysis and perineum can be infiltrated with local anaesthesia in anticipation of a possible emergency symphysiotomy and episiotomy. If there is no progress after 30–60 minutes of bearing down, a symphysiotomy is performed. The threshold for symphysiotomy in breech presentations should not be too high because it is not easy to predict which baby will otherwise die and one wants to prevent damaged babies as well as fatalities.

This policy will, in my experience, together with the more or less mandatory CSs in certain breech presentations (abnormal pelvis, oxytocin-resistant arrest in first stage of labour, placenta praevia, (impending) eclampsia after stabilisation, bad obstetric history, high parity plus sterilisation requested [14], and/or HIV-positive status, etc.) result in an overall 20% CS rate in breeches (most with both maternal and foetal indications) and a somewhat less than 5% symphysiotomy rate.

The ministries of health in resource-challenged countries should encourage vacuum extraction for delay in second stage of labour. They should not accept the argument, used by the staff of some obstetric departments, that the procedure causes abrasions on the scalp and therefore more vertical HIV transmission, because so does waiting for a CS. Besides, there are other ways to prevent HIV transmission, including virucidal lubricants on the cup. Failed vacuum extractions with live babies should be resolved by symphysiotomies.

One should ensure that symphysiotomy is included in the national obstetrical curricula, at least as long as cases of VVF directly due to obstructed labour are seen. The World Health Organization could present a “VVF Free Certificate” to a country if these fistulas have disappeared, after which medical schools would perhaps have some reason to stop the clinical training in symphysiotomies.

The development and proper marketing of a sophisticated, affordable non-disposable instrument for symphysiotomies would assist the obstetricians of Africa in revisiting this operation, which has not been modified for nearly a hundred years.

## Discussion

Björklund's meta-analysis of 5,000 symphysiotomies [1] showed that a woman who had a symphysiotomy did better than a woman who had a CS in relation to the index delivery, as well as in future deliveries when she would have a larger pelvis and no uterine scar. However, this is an inaccurate comparison for the circumstances prevailing in many hospitals in Africa. The meta-analysis concerned studies performed in conditions where performing an elective CS for breech presentation or doing an emergency CS when an instrumental delivery was feasible were considered strong evidence of mismanagement. In contrast, in sub-Saharan Africa presently, not performing a CS for these indications is often seen by the regional obstetrical establishment as misguided. Under the latter circumstances, to be fair, an evaluation should—in nullipara—compare the combined complications (and financial costs) of between 25 CSs (reintroducing vacuum extractions, symphysiotomy in case of failure) to 42 CSs (breeches, see Table 1) plus many trials of scar, with those of one symphysiotomy. Many CSs can be prevented not by performing symphysiotomies but by reintroducing them as an acceptable option. When that policy change has occurred then it becomes reasonable, in the individual case during delivery, to compare the expected side effects of one CS with those of one symphysiotomy, but only when one is certain that one is dealing with the last delivery of the woman involved.

Currently, the advantages and disadvantages of preferring one CS above one symphysiotomy are not known because Björklund could not analyse the results on an intention-to-treat basis [1]. If, for example, delivery was obstructed in (nearly) the second stage of labour and the foetus died in the theatre queue, then an alert doctor would often have cancelled the CS and have performed a craniotomy, or the associated moulding might well have resulted in a spontaneous delivery. The infection and/or mechanical pressure implicated in the foetal demise could also affect the mother and can lead to metritis, peritonitis, sepsis, infertility, uterine rupture, and/or the development of a fistula, but neither these nor the foetal death would then have been recorded as CS related. On the other hand some women deliver a live baby in the theatre queue after a failed vacuum extraction without a symphysiotomy.

The data produced by this analysis are so robust that the basic idea of this essay stands, even if the assumptions can be challenged to some extent.

## Conclusion

Calculations (using the assumptions in Box 2) show in relation to breech deliveries in nullipara (see Table 1) that 356 (1.96/0.0055) times as many symphysiotomies have to be performed in rich countries as in resource-deprived countries, to prevent the death of one woman. To have one extra baby survive, 185 (1.48/0.008) times as many symphysiotomies have to be performed.

A policy of reintroducing vacuum extractions combined with performing symphysiotomies in cases of failure is 3,500 (10,500/3) times more effective in terms of preventing maternal deaths per symphysiotomy in resource-challenged countries, in nullipara, and 1,785 (7,140/4) times as useful in preventing perinatal and infant deaths (see above under vacuum extractions). Additionally, the opportunity to perform a symphysiotomy for obstetricians in resource-rich countries is so rare and urgent that an acceptable level of expertise cannot be attained.

These dissimilarities in the obstetrical environments have to have policy implications. The refutation: “If symphysiotomies are such good operations why don't you perform them at home?” has therefore little merit.

There is at the very least enough evidence to start—for cases of arrest and/or foetal distress in the second stage of labour—a randomised long-term cohort study comparing (1) vacuum extractions on occasion combined with a symphysiotomy with (2) a slot in the theatre queue.

Another study could compare the long-term results of hospitals using (1) CSs and episiotomies to prevent or solve all mechanical labour problems with (2) the use of more options including CSs, symphysiotomies, craniotomies, fundal pressure, oxytocin according to a proper protocol, and vacuum extractions. Women in Nigeria may well be willing to participate in such studies.

## Abbreviations

CS: caesarean section
TFR: total fertility rate
VVF: vesico-vaginal fistula
